# Total Phenolic Content-Based Optimization of Ultrasound-Assisted Extraction from *Eupatorium cannabinum* L. Aerial Parts and In Vitro Evaluation of the Resulting Extract

**DOI:** 10.3390/plants15213277

**Published:** 2026-10-23

**Authors:** Mihai Babotă, Nastaca Alina Palade, Ingrid Nădășan, Alexandru Nicolescu, Bianca-Alisa Dragnea, Ruxandra Ștefănescu, Andrei Mocan, Oleg Frumuzachi, Adrian Man, Lénárd Farczádi, Corneliu Tanase

**Affiliations:** 1 Department of Pharmaceutical Botany, Faculty of Pharmacy, “George Emil Palade” University of Medicine, Pharmacy, Science, and Technology of Târgu Mureș, 38 Gheorghe Marinescu Street, 540139 Târgu Mureș, Romania; mihai.babota@umfst.ro (M.B.); bianca13alisa@yahoo.com (B.-A.D.); corneliu.tanase@umfst.ro (C.T.); 2 Research Center of Medicinal and Aromatic Plants, “George Emil Palade” University of Medicine, Pharmacy, Science, and Technology of Târgu Mureș, 38 Gheorghe Marinescu Street, 540139 Târgu Mureș, Romania; ingrid.nadasan@umfst.ro; 3 Preclinical Department, Faculty of Medicine, “Lucian Blaga” University of Sibiu, 2A Lucian Blaga Street, 550169 Sibiu, Romania; 4 Doctoral School of Medicine and Pharmacy, “George Emil Palade” University of Medicine, Pharmacy, Science, and Technology of Târgu Mureș, 38 Gheorghe Marinescu Street, 540139 Târgu Mureș, Romania; 5 Laboratory of Chromatography, Institute of Advanced Horticulture Research of Transylvania, University of Agricultural Sciences and Veterinary Medicine, 3–5 Calea Mănăștur, 400372 Cluj-Napoca, Romania; alexandru.niculescu@itim-cj.ro (A.N.); mocan.andrei@umfcluj.ro (A.M.); 6 National Institute for Research and Development of Isotopic and Molecular Technologies, 67-103 Donat Street, 400293 Cluj-Napoca, Romania; 7 Department of Pharmacognosy and Phytotherapy, Faculty of Pharmacy, “George Emil Palade” University of Medicine, Pharmacy, Science, and Technology of Târgu Mureș, 38 Gheorghe Marinescu Street, 540139 Târgu Mureș, Romania; ruxandra.stefanescu@umfst.ro; 8 Department of Pharmaceutical Botany, Faculty of Pharmacy, “Iuliu Hațieganu” University of Medicine and Pharmacy, 23 Gheorghe Marinescu Street, 400337 Cluj-Napoca, Romania; oleg.frumuzachi@elearn.umfcluj.ro; 9 Department of Microbiology, Faculty of Medicine, “George Emil Palade” University of Medicine, Pharmacy, Science, and Technology of Târgu Mureș, 38 Gheorghe Marinescu Street, 540139 Târgu Mureș, Romania; adrian.man@umfst.ro; 10 Chromatography and Mass Spectrometry Laboratory, Center for Advanced Medical and Pharmaceutical Research, “George Emil Palade” University of Medicine, Pharmacy, Science, and Technology of Târgu Mureș, 38 Gheorghe Marinescu Street, 540142 Târgu Mureș, Romania; lenard.farczadi@umfst.ro

**Keywords:** *Eupatorium cannabinum*, ultrasound-assisted extraction, design of experiments, total phenolic content, antioxidant potential, enzyme inhibition, antibacterial activity

## Abstract

*Eupatorium cannabinum* L. (Asteraceae) is a medicinal plant with incompletely characterized phytochemical and biological properties. This study optimized ultrasound-assisted extraction (UAE) of phenolic compounds from its aerial parts, using total phenolic content (TPC) as the only response, and evaluated the extract in exploratory in vitro assays. A D-optimal design assessed the effects of extraction time, ultrasound amplitude, and ethanol concentration. The regression model showed good fit and predictive ability (R^2^ = 0.979, Q^2^ = 0.798), and ethanol concentration was the dominant factor. The optimum (30% ethanol, 20 min, 40% amplitude) was confirmed experimentally, yielding 31.32 ± 0.58 mg GAE/g dried plant in the liquid extract. The lyophilized optimized extract (OpEU), analyzed separately, contained 64.64 ± 2.64 mg GAE/g dry extract; because the two values are expressed on different mass bases (dried plant vs. dry extract), they are not directly comparable. Luteolin-7-*O*-glucoside and ellagic acid were the main targeted phenolics in OpEU (UHPLC-PDA). OpEU scavenged DPPH and ABTS radicals (IC_50_ of 5.59 and 0.83 mg dry extract/mL) and weakly inhibited α-glucosidase, α-amylase, and pancreatic lipase (IC_50_ of 3.031, 3.553, and 2.665 mg dry extract/mL). It inhibited *Staphylococcus aureus*, methicillin-resistant *S. aureus*, *Escherichia coli*, and *Klebsiella pneumoniae* only at the highest concentration tested (5.09 mg/mL), but not *Enterococcus faecalis* or *Pseudomonas aeruginosa*, and it was not bactericidal. These preliminary results do not support health-related applications, which would require pyrrolizidine alkaloid and toxicological evaluation.

## 1. Introduction

*Eupatorium cannabinum* L. (hemp agrimony) is a perennial species of the Asteraceae family with a documented ethnopharmacological history [1,2]. Its secondary metabolites include flavonoids such as rutin and quercetin, sesquiterpene lactones such as eupatoriopicrin and eucannabinolide, and pyrrolizidine alkaloids such as echinatine and supinine [3,4]. Although some of these constituents have been linked to pharmacological activity, pyrrolizidine alkaloids present toxicological concerns and therefore biological activity alone cannot be taken as evidence that an *E. cannabinum* preparation is safe or suitable for pharmaceutical or nutritional use.

Several activities have been reported for *E. cannabinum* extracts and their individual constituents. For example, an ethanolic extract inhibited several bacterial strains, particularly the Gram-positive *Enterococcus faecalis*, and the volatile oil was reported to be highly active against the resistant strain *Pseudomonas aeruginosa* [5]. Other studies mostly evaluated cytotoxicity. Eupatoriopicrin, the main sesquiterpene lactone, was cytotoxic to human and animal cancer cells in culture and was associated with DNA damage in Ehrlich ascites carcinoma cells [6]. It also showed cytostatic effects and inhibited tumor growth in Lewis lung carcinoma and fibrosarcoma models [7]. Furthermore, Beekman et al. compared the cytotoxicity of eupatoriopicrin against a panel of tumor cell lines, including murine EAT, P388, FIO 26 and L5178Y(s) cells and human HeLa cells [8]. Ribeiro-Varandas et al. showed that an ethanolic extract reduced the viability of HT29 colon cancer cells [7]. Ionita et al. reported antiproliferative effects of a fractionated hydroethanolic extract on BT-20, HepG2, Caco-2 and Jurkat cells, with both polar and non-polar fractions contributing, but the compounds and mechanisms responsible remained unidentified [5]. Lastly, Abuali et al. found that *E. cannabinum* was cytotoxic to MCF-7 breast cancer cells and identified eucannabinolide as an inducer of apoptosis (annexin V/PI assay) [9]. Because cytotoxic activity cannot establish safe exposure levels, these findings also underline the need for dedicated safety assessment of *E. cannabinum* preparations.

Previous work has focused on the in vitro biological characterization of hydroalcoholic extracts, fractions, essential oils and isolated constituents [5,6,7,8,9]. The comparability of the results is difficult due to differences in solvents, extraction procedures, and selected biological models. To our knowledge, the effect of extraction conditions on the recovery of phenolic compounds from *E. cannabinum* has not been studied with an experimental design approach that considers extraction time, solvent concentration, and ultrasound amplitude together, and such approaches have been only applied to other *Eupatorium* species. For example, the ultrasound-assisted extraction of total alkaloids from *E. lindleyanum* was optimized using a Box–Behnken design that varied extraction time, temperature, and liquid-to-solid ratio [10].

The main objective of this study was therefore to optimize the ultrasound-assisted extraction (UAE) of phenolic compounds from the aerial parts of *E. cannabinum*, using total phenolic content (TPC) as the sole response variable. A design of experiments approach was used to evaluate the effects of extraction time, ultrasound amplitude, and ethanol concentration. The resulting TPC-optimized freeze-dried extract (OpEU) was then characterized by UPLC-PDA and screened in vitro for antioxidant, enzyme-inhibitory, and antibacterial activity. The study did not include pyrrolizidine alkaloid profiling or any cytotoxicity, genotoxicity, or other toxicological testing, so no conclusions about the safety or clinical use of the extract could be drawn.

## 2. Results and Discussion

### 2.1. Histo-Anatomical Analysis of E. cannabinum

Microscopic examination was carried out to document the diagnostic features of the aerial parts used for extraction. Cross-sections of the leaf, petiole, and stem were double-stained to distinguish cellulosic walls (pink-red) from lignified walls (green-blue). Together with the voucher specimen (EUP0622), these features document the identity of the plant material and can serve as a reference for pharmacognostic identification.

**Figure 1:**
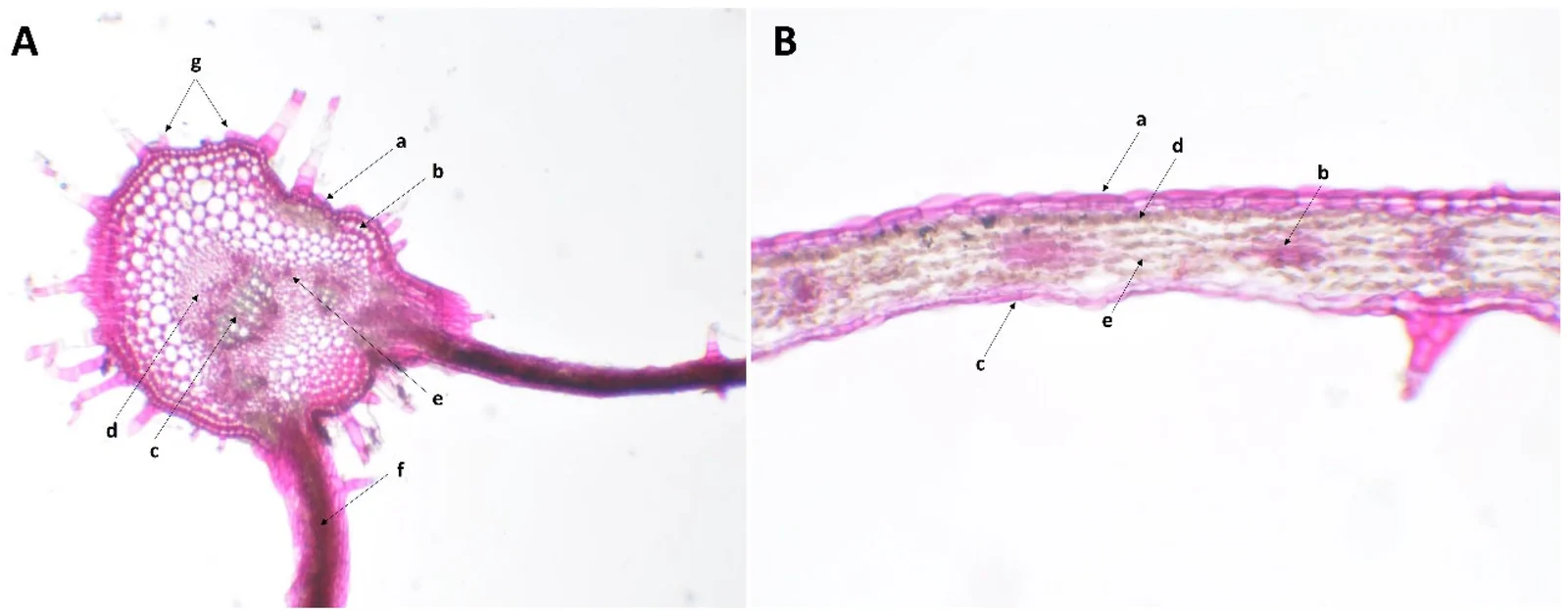
Cross-sections of the *E. cannabinum* L. leaf. (**A**) Midrib (4× magnification): a—epidermis; b—cortex; c—xylem; d—phloem; e—collenchyma; f—leaf blade (lamina); g—multicellular covering trichomes and protective layer. (**B**) Lamina (10× magnification): a—upper epidermis; b—vascular bundle; c—lower epidermis; d—palisade tissue; e—spongy tissue. Double staining: ruthenium red (cellulosic walls, pink-red) and iodine green (lignified walls, green-blue).

The leaf structure of *E. cannabinum* can be described as bifacial. In cross-section through the midrib (Figure 1A), the leaf is bounded by an upper and a lower epidermis of tightly packed cells. Multicellular covering trichomes occur along the midrib surface. The midrib has a parenchymatous cortex with a central vascular system and collenchyma. Through the lamina (Figure 1B), the mesophyll is differentiated into palisade and spongy tissue, and the lamina is thin, with small vascular bundles.

**Figure 2:**
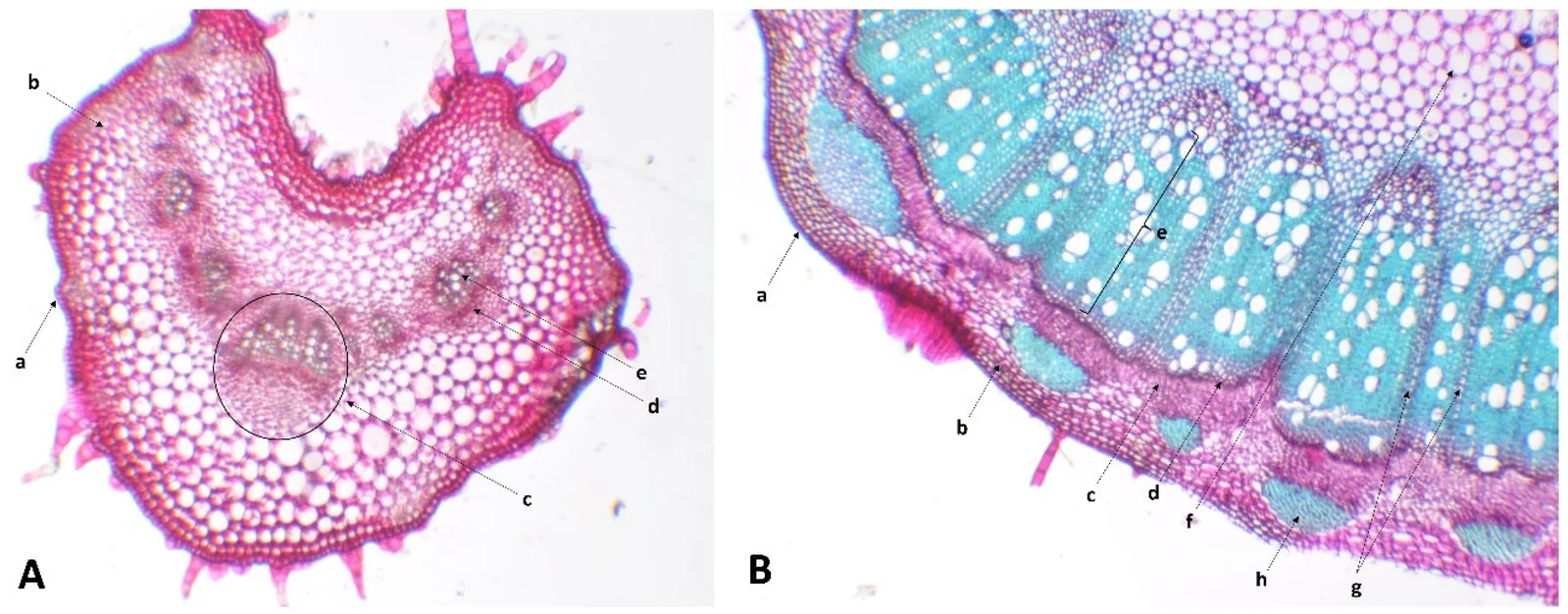
Cross-sections of *E. cannabinum* L. (**A**) Petiole (4× magnification): a—epidermis; b—cortex; c—collateral vascular bundle; d—sclerenchyma; e—medullary rays. (**B**) Stem (10× magnification): a—epidermis; b—primary cortex; c—phloem; d—cambium; e—secondary xylem; f—medullary parenchyma; g—medullary rays; h—sclerenchyma. Double staining: ruthenium red (cellulosic walls, pink-red) and iodine green (lignified walls, green-blue).

In the anatomical structure of the petiole (Figure 2A), the first visible component is the epidermis, containing isodiametric cells with a thickened wall and bearing sporadic multicellular covering trichomes. Beneath it lies a cortex, and the vascular tissue consists of collateral bundles arranged in an arc, associated with the sclerenchyma. The stem (Figure 2B) shows an epidermis, a primary cortex, and a central cylinder. The central cylinder contains a phloem ring and a lignified secondary xylem ring separated by a cambium, with medullary rays crossing the xylem. Sclerenchyma lies outside the phloem, and the center is occupied by medullary parenchyma.

### 2.2. Experimental Design of UAE and Data Fitting

In this study, extraction time, ultrasound amplitude, and ethanol concentration were chosen as factors on the basis of the UAE mechanism, the equipment used, and the nature of the plant material. UAE acts mainly through cavitation at the interface between the solvent system and the plant particles, which disrupts the plant tissue, reduces particle size, and increases the contact surface between solvent and matrix. It also ruptures cell walls, which releases bioactive compounds stored inside the cells, especially in vacuoles [11,12]. The aerial parts of herbaceous plants are fragile and, once the lignified stems are removed, they contain few strongly lignified tissues. We therefore used moderate ranges of extraction time (5–20 min) and amplitude (20–40%), which also limited energy consumption and wear on the equipment. An ethanol–water mixture (30–70% ethanol, *v*/*v*) was chosen as the solvent because its intermediate polarity is suitable for the extraction of the target compounds, the total polyphenols [13,14].

The D-optimal-based DoE comprised 16 runs, including three center points (Table 1). TPC ranged from 11.41 to 30.72 ± 1.38 mg GAE/g of dried plant. The highest value was obtained with 30% ethanol, 20 min, and 40% amplitude, corresponding to run N4. Generally, it was observed that TPC increased as ethanol concentration decreased and extraction time increased, while amplitude had a smaller effect.

Energy input behaved in a different manner: it depended mainly on amplitude and extraction time, rising from 40 to 47 Ws (5 min, 20% amplitude) to 2399 to 2493 Ws (20 min, 40% amplitude), and it was unaffected by ethanol concentration. The highest observed TPC (N4) was therefore the most energy-demanding. For comparison, 30% ethanol, 20 min and 20% amplitude (N2) yielded 26.89 mg GAE/g plant at 185 Ws, about 13 times less energy for about 12% less TPC.

### 2.3. Model Fitting and Influence of Process Parameters

**Figure 3:**
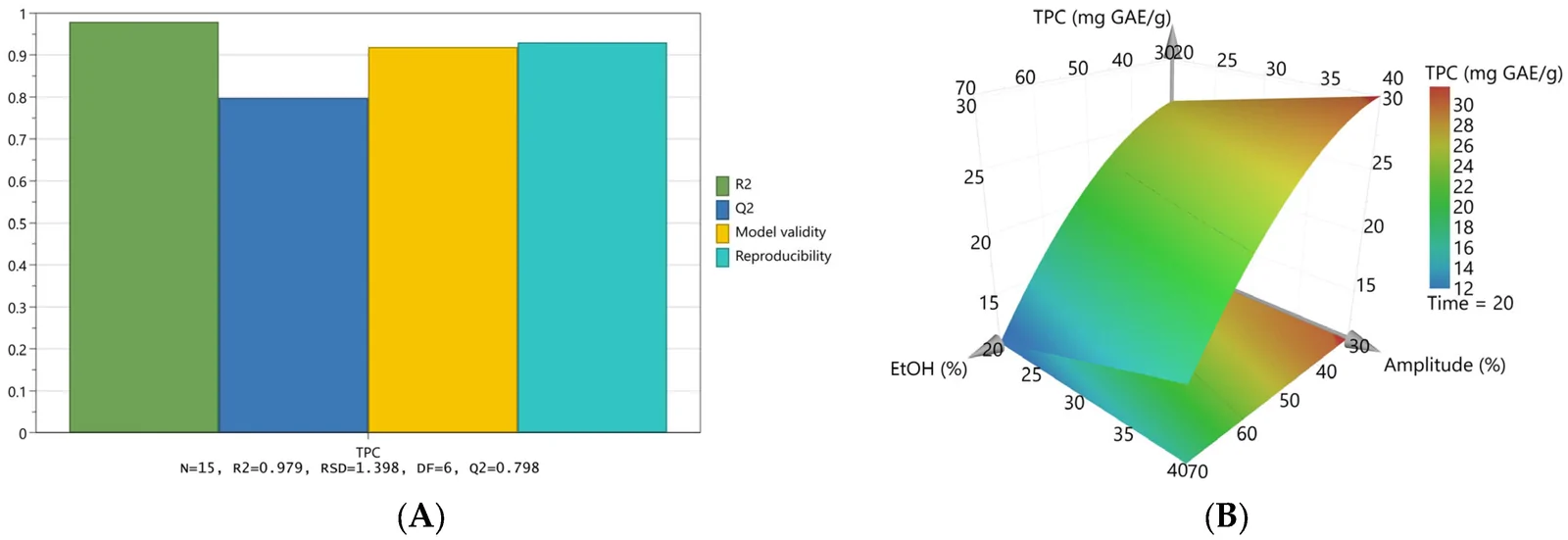
(**A**) Summary of fit of the regression model for total phenolic content (TPC): R^2^ (goodness of fit, 0.979), Q^2^ (goodness of prediction, 0.798), model validity (0.919), and reproducibility (0.930). Values close to 1 indicate a good model; model validity > 0.25 indicates no significant lack of fit, and reproducibility > 0.5 indicates low variability among the replicated center points. (**B**) Response surface plot (RSP) showing the predicted TPC (mg GAE/g dried plant) as a function of ethanol concentration (30–70%, *v*/*v*) and ultrasound amplitude (20–40%), with the extraction time held constant at 20 min. The color scale indicates predicted TPC values, from lowest to highest.

The TPC data from the 16 runs were fitted by regression in MODDE using an interaction and quadratic model. The model summary (Figure 3A) indicated a good fit and predictive ability, characterized by R^2^ = 0.979, adjusted R^2^ = 0.951, Q^2^ = 0.798, model validity = 0.919, and reproducibility = 0.930 [14,15]. The R^2^ and adjusted R^2^ values indicated that the model explains the experimental data well, and Q^2^ showed good predictive power. The validity value indicates no significant lack of fit, and the reproducibility value reflects the low variability among the three center points (see also Supplementary Materials).

**Figure 4:**
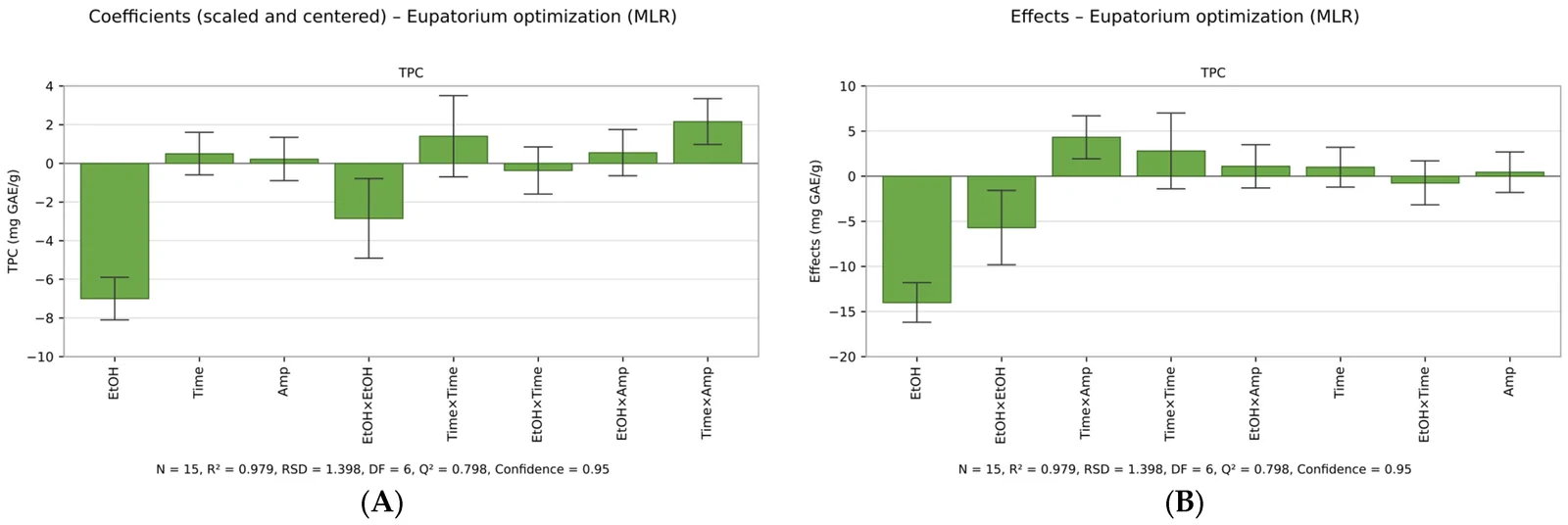
Graphical model interpretation. (**A**) Scaled and centered coefficients of the regression model, showing the effects of ethanol concentration, extraction time, and ultrasound amplitude, and their interactions, on TPC. (**B**) Effects of these parameters on TPC presented in decreasing order of their magnitude.

Subsequently, the effects of the factors on TPC were analyzed and are shown in Figure 3B and Figure 4. Ethanol concentration was the dominant factor and had a strongly negative effect (*p* = 4.3 × 10^−6^): the lowest TPC values (11.41–16.12 mg GAE/g dried plant) were all obtained with 70% ethanol, and the highest with 30% ethanol (Table 1). This may reflect the better solubility of polar phenolic compounds in highly polar and hydrophilic solvents, although this was not tested specifically. Extraction time had a smaller, positive effect, and ultrasound amplitude had the smallest overall effect, with the advantage of longer extraction mainly observed at low ethanol concentration. At 30% ethanol, extending the time from 5 to 20 min increased TPC by about 6–7 mg GAE/g, whereas at 70% ethanol the effect was small and inconsistent. The most significant terms besides the linear ethanol effect were the time × amplitude interaction (coefficient +2.16; 95% CI 0.97 to 3.35; *p* = 0.004) and the ethanol-squared term (coefficient −2.85; 95% CI −4.91 to −0.79; *p* = 0.015).

Within the investigated range of 20–40%, higher amplitude did not reduce TPC consistently, which was also observed in the response surface plot in Figure 3B. The results are consistent with prior research, which has indicated that high-intensity ultrasound frequently has an adverse effect on the release of polyphenols and may be related to an alteration in their chemical composition [16,17].

### 2.4. Design Space and TPC-Based Process Optimization

Once the experimental model was considered adequate, it was used to find the conditions that maximize TPC within the investigated domain. Based on the optimized function (with the goal of maximizing TPC), the predicted optimum was 30% ethanol, an extraction time of 20 min, and an ultrasound amplitude of 40% (Table 2), which coincides with run N4, the highest-yielding run of the design (Table 1). Under these conditions, the model predicted 30.27 mg GAE/g plant, and the confirmation experiment yielded 31.32 ± 0.58 mg GAE/g plant (*n* = 3), on average 3.5% above the predicted value (the ±range reflects only the variability between the three confirmation extractions). This close agreement supported the predictive ability of the model.

This optimum lies at the boundary of the design space for all three factors: the lowest ethanol concentration and the highest time and amplitude tested. Higher TPC might therefore be reached outside the investigated ranges, for example, with less than 30% ethanol or longer extraction. For this reason, the conditions represent the optimal only within the studied domain, and only for TPC as response. Since no conventional extraction method or alternative UAE condition was evaluated, the study does not imply that these conditions are superior for biological activity or for the recovery of individual compounds.

In order to obtain a significant mass for biological analyses, the optimized UAE was repeated 25 times (25 × 0.5 g of plant material). Lastly, all repeated extractions were pooled and lyophilized, obtaining 1.64 g of dry extract (OpEU), corresponding to an extraction yield of 14.26% (*m*/*m*).

### 2.5. Total Phenolic Content of the Optimized Extract (OpEU)

The TPC of OpEU, determined after lyophilization, was 64.64 ± 2.64 mg GAE/g (Table 3). This value cannot be compared directly with the 31.32 mg GAE/g dried plant obtained in the confirmation run, as the two are expressed on different bases. Because the two values were obtained on different materials (liquid extracts vs. the final lyophilized powder) and expressed on different mass bases, they cannot be used to calculate a mass balance or recovery, and no such balance was established in this study. The TPC of OpEU should therefore be read as the content of the final powder, not of the liquid extracts used in the optimization.

Only a limited number of studies have specifically investigated the total phenolic content of *E. cannabinum*. Dutta and Mahanta reported a TPC value of 64.82 mg GAE/g of dry mass for the methanol extract of leaves originating in Assam, India [3]. For another species of the genus, leaf extracts of *E. adenophorum* contained 30.71 ± 0.16 to 68.51 ± 0.58 mg GAE/g of sample, depending on the solvent [18]. The OpEU value is therefore in the upper part of that range. Direct comparison is limited because TPC is highly dependent on species and plant organ, growing conditions, developmental stage, solvent, extraction method, solid-to-solvent ratio, and expression units (either dry plant or dry extract).

### 2.6. Radical-Scavenging Antioxidant Activity

The radical-scavenging activity of OpEU was assessed using the standard in vitro DPPH and ABTS assays, which are widely used, complementary chemical tests that measure the ability of an extract to neutralize stable synthetic radicals [19]. However, they do not reproduce biological oxidative processes, and the presented results only indicate a chemical reducing capacity.

**Figure 5:**
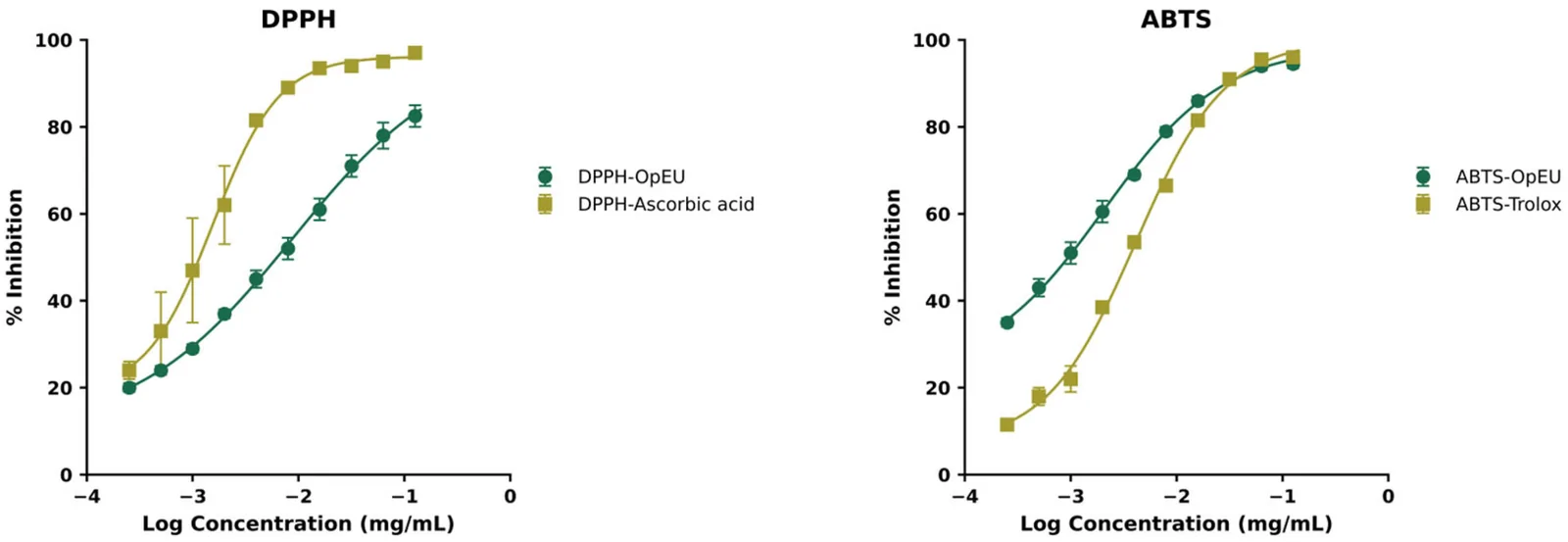
Concentration-dependent scavenging of the ABTS and DPPH radicals by OpEU, presented as logarithmic inhibition curves (log of concentration plotted against the percentages of inhibition). Data are expressed as mean ± SD of three technical replicates from one pooled extract batch. IC_50_ values for reference compounds were ascorbic acid (DPPH) 0.0037 ± 0.0004 mg/mL; Trolox (ABTS) 0.0015 ± 0.0001 mg/mL.

It was observed that OpEU scavenged both radicals in a concentration-dependent manner (Figure 5), with IC_50_ values of 5.59 ± 0.68 mg dry extract/mL (95% CI 3.90–7.28) in the DPPH assay and 0.83 ± 0.11 mg dry extract/mL (95% CI 0.56–1.10) in the ABTS assay (Table 3). Under these assay conditions, the IC_50_ obtained in the ABTS assay was about seven times lower than that obtained in the DPPH assay. The two radicals differ in chemical nature, solvent system, reaction kinetics, and accessibility to the extract constituents [20]. Since these factors were not tested experimentally, no scavenging mechanism can be inferred.

The reference compounds were much more active: ascorbic acid had an IC_50_ of 0.0037 ± 0.0004 mg/mL in the DPPH assay and Trolox 0.0015 ± 0.0001 mg/mL in the ABTS assay. The IC_50_ of OpEU was therefore about 1500 times (DPPH) and 540 times (ABTS) higher than the references, yet this comparison should be interpreted with caution, since OpEU is a complex mixture and the references are pure compounds.

Previously, Ionita et al. reported a DPPH IC_50_ value of 2.91 mg/mL for a hydroethanolic extract of *E. cannabinum* [5], which is about half of the DPPH IC_50_ obtained for OpEU (5.59 mg dry extract/mL). Differences in plant material, extraction conditions, composition, and assay protocol further limit this comparison. Electrochemical methods have also shown antioxidant properties for *E. cannabinum* extracts [2], but results from different analytical principles cannot be compared directly with DPPH or ABTS IC_50_ values. Moreover, only the OpEU was tested, so the data cannot indicate how TPC relates to antioxidant activity, nor what recovered secondary metabolites caused the effect.

### 2.7. Enzyme Inhibitory Activity of OpEU

OpEU inhibited α-glucosidase, α-amylase, and pancreatic lipase in vitro in a measurable manner (Table 4), with the IC_50_ values being 3.031 ± 0.095 mg/mL for α-glucosidase, 3.553 ± 0.334 mg/mL for α-amylase, and 2.665 ± 0.356 mg/mL for lipase. Thus, the extract was slightly more active against lipase than against the two carbohydrate-hydrolyzing enzymes. The positive controls were much more potent, with IC_50_ values of 0.155 ± 0.021 mg/mL (acarbose, α-glucosidase), 0.021 ± 0.001 mg/mL (acarbose, α-amylase) and 0.068 ± 0.002 mg/mL (orlistat, lipase). The IC_50_ of OpEU was therefore about 20, 170, and 40 times higher, respectively, so the extract showed weak inhibitory activity under the investigated conditions [20]. As with the antioxidant assays, the comparison is limited because OpEU is a complex mixture while the standard experimental controls are pure compounds.

Data on the enzyme-inhibitory activity of *E. cannabinum* are scarce, and based on our literature search, no previous report exists for this species. Other members of the genus have been studied for metabolic targets; for example, flavonoids from *E. lindleyanum* were evaluated for enzyme-inhibitory activity [21], and germacrane-type sesquiterpenes from *E. chinense* inhibited protein tyrosine phosphatase, an enzyme involved in insulin signaling [21]. These results are preliminary biochemical observations and, in the absence of any cellular or in vivo data, do not establish antidiabetic or anti-obesity activity. The extract was not optimized for these activities, and therefore the activity cannot be assigned to individual constituents.

### 2.8. Antibacterial Activity of OpEU

The antibacterial activity of OpEU was assessed by broth microdilution against three Gram-positive strains (*S. aureus* ATCC 25923, MRSA ATCC 43300, and *E. faecalis* ATCC 29212) and three Gram-negative strains (*E. coli* ATCC 25922, *K. pneumoniae* ATCC 13883, and *P. aeruginosa* ATCC 27853). The minimum inhibitory concentration (MIC) was defined as the lowest tested concentration at which no growth was detected, and the minimum bactericidal concentration (MBC) as the lowest concentration giving no colony growth after subculture on agar (Table 5).

OpEU inhibited the growth of *S. aureus*, MRSA, *E. coli*, and *K. pneumoniae* only at the highest tested concentration (5.09 mg/mL). For *E. faecalis* and *P. aeruginosa*, no inhibitory endpoint was reached (MIC > 5.09 mg/mL). The MBC was above 5.09 mg/mL for all strains, so no bactericidal effect was detected in the range tested. The pattern did not follow the Gram type: inhibition occurred in both Gram-positive (*S. aureus*, MRSA) and Gram-negative (*E. coli*, *K. pneumoniae*) strains, while one strain of each type (*E. faecalis*, *P. aeruginosa*) was unaffected. The positive control, levofloxacin, was active against all strains at concentrations about four orders of magnitude lower (0.03–0.5 μg/mL). Overall, it can be considered that OpEU showed weak growth-inhibitory activity and no bactericidal activity under these experimental conditions.

Antibacterial effects have been reported for other *E. cannabinum* preparations. Purcaru et al. described the activity of chloroform and hydroalcoholic extracts against *E. coli* and *Bacillus cereus* [22], while Senatore et al. reported that the essential oil from the aerial parts acted mainly on Gram-positive bacteria, including *S. aureus* [23]. Ionita et al. reported an ethanolic extract active particularly against *E. faecalis* [5], whereas OpEU was inactive against this strain, and the essential oil, a volatile and chemically different preparation, cannot be compared with this polar extract. Differences in solvent, extraction method, composition, strains, and test conditions may explain the discrepancies. In our case, the extraction conditions were chosen only to maximize TPC, not antibacterial activity, so a high TPC does not imply strong activity. In any case, the data do not support the use of OpEU as a natural antimicrobial, and whether other extraction conditions, fractions, or isolated constituents are more active is the subject of further studies.

### 2.9. UPLC-PDA Phenolic Profiling

OpEU was qualitatively and quantitatively analyzed by targeted UHPLC-PDA for twelve phenolic compounds (flavonoid derivatives and phenolic acids) for which reference standards were available. Compounds were identified by comparing retention times and UV spectra with those of the standards and quantified at the wavelength giving the best selectivity and sensitivity for each analyte (Table 6; chromatograms presented in Supplementary Materials, Figure S1).

All twelve compounds were detected in OpEU. Six of them (eleutheroside B, epicatechin, caffeic acid, vanillic acid, taxifolin and sinapic acid) were below the limit of quantification (LoQ) and are therefore only reported as detected. The targeted profile was dominated by luteolin-7-*O*-glucoside (67.4 mg/g dry extract) and ellagic acid (28.6 mg/g), followed by quercetin (8.7 mg/g) and rutin (8.3 mg/g), and lastly, catechin (2.0 mg/g) and chlorogenic acid (2.3 mg/g) were minor constituents. Rutin and quercetin have also been reported in a 70% hydroethanolic extract of the aerial parts prepared by maceration [5], and the solvent used was shown to influence the phytochemical profile of *E. cannabinum* extracts [24].

### 2.10. Limitations and Safety Considerations

An important limitation of the present study is the absence of a dedicated safety assessment. Species belonging to the genus *Eupatorium* may contain pyrrolizidine alkaloids, which require toxicological consideration. The UHPLC-PDA method was targeted for twelve phenolic compounds and was not designed to detect or quantify these alkaloids, so their presence and/or concentration in OpEU cannot be established from our data.

Furthermore, OpEU was not evaluated using cytotoxicity, genotoxicity, or other toxicological assays. Consequently, the in vitro antioxidant, enzyme-inhibitory, and antibacterial findings should not be interpreted as evidence of safety, therapeutic efficacy, or suitability for pharmaceutical, nutritional, or health-promoting applications. Before any such application can be considered, future studies should include targeted profiling and quantification of relevant pyrrolizidine alkaloids using an appropriately validated analytical method, together with cytotoxicity, genotoxicity, and other relevant safety assessments.

Regarding the optimization design, TPC was the only response variable, so the obtained conditions are optimal only within the experimental ranges (30–70% ethanol, 5–20 min, 20–40% amplitude). They represent the boundary of this domain, so higher TPC may be reachable outside it. No conventional extraction method or other UAE condition was run as a comparator, so the study does not show that the optimized conditions are superior in the recovery of individual compounds or for any biological activity.

## 3. Materials and Methods

### 3.1. Chemicals and Reagents

The chemical substances used in the extraction and in vitro determinations included the following: 95% ethanol (Girelli Alcool Srl, Zibido, San Giacomo, Italy), 99.5% sodium carbonate decahydrate (Reactivul SRL, Râmnicu Vâlcea, Romania), and 2N (2 mol/L) Folin–Ciocâlteu reagent (Merck KGA, Darmstadt, Germany). For antioxidant activity, 2,2-diphenyl-1-picrylhydrazyl (DPPH, ≥98%) and 2,2′-azino-bis(3-ethylbenzothiazoline-6-sulfonic) (ABTS, ≥98%) were used, and these were acquired from Sigma-Aldrich Chemie GmbH (Steinheim, Germany). For enzymatic activity, α-glucosidase (G5003, ≥10 units/mg protein), α-amylase (A3176, ≥5 units/mg solid), and lipase (L3126, ≥125 units/mg protein) were used, and these were purchased from Sigma-Aldrich, Saint Louis, MO, USA.

The antibacterial activity was tested against 6 different bacterial strains: *Staphylococcus aureus* ATCC 25923, Methicillin-resistant *Staphylococcus aureus* (MRSA) ATCC 43300, *E. faecalis* ATCC 29212, *Escherichia coli* ATCC 25922, *Klebsiella pneumoniae* ATCC 13883, and *Pseudomonas aeruginosa* ATCC 27853. These strains were provided by the Microbiology Department of the “George Emil Palade” University of Medicine, Pharmacy, Science, and Technology of Târgu Mureș.

### 3.2. Plant Material

The aerial parts of *Eupatorium cannabinum* L. were harvested in July 2022 from the banks of the Mureș River, Sîntana de Mureș village, Mureș County, Romania (46°33′51″ N, 24°33′59″ E, 320 m above sea level). The plant was identified by Prof. Corneliu Tanase, and a voucher specimen (EUP0622) was deposited in the Herbarium of the Pharmaceutical Botany Department, “George Emil Palade” University of Medicine, Pharmacy, Science and Technology of Târgu Mureș. The plant collection complied with institutional, national, and international guidelines and legislation. Subsequently, the material was dried at room temperature, protected from light, for a total of 10 days, and stored at the Research Center of Medicinal and Aromatic Plants in Târgu Mureș until the extraction phase. Before extraction, the lignified stems were manually removed from the product, achieved by appropriate crushing or grinding.

### 3.3. Histo-Anatomic Analysis

Transverse sections of the leaf, petiole, and stem were cut by hand from dried and rehydrated material of one representative specimen. The sections were double-stained with iodine green (1% aqueous solution acidified with 2% acetic acid), which stains lignified walls green-blue, and ruthenium red (1% aqueous solution), which stains cellulosic walls pink-red [25]. Five sections from each organ were examined using a Motic B-series light microscope (Motic China Group Co., Ltd., Xiamen, China) and photographed with a Nikon Coolpix L22 camera (Nikon Corporation, Tokyo, Japan). The micrographs shown are representative of the features observed.

### 3.4. Ultrasound-Assisted Extraction and Design of Experiments

For the extraction procedure, the dried plant material was finely ground using a Pulverisette 15 cutting mill (Fritsch GmbH, Idar-Oberstein, Germany) and passed through a 1 mm sieve according to Ph. Eur. 10.6, to ensure particle size uniformity. The extractions were performed using the ultrasonic probe-type disruptor UP200St (200 W, 26 kHz; Hielscher Ultrasonics GmbH, Teltow, Germany), equipped with an immersion probe or sonotrode of type S26d2 (2 mm diameter, 120 mm length, 2–50 mL capacity). In each run, 0.5 g of plant powder was suspended in 10 mL of an ethanol-in-water mixture (% *v*/*v*, prepared from 95% ethanol). The suspension was sonicated in an ice bath to limit heating and then vacuum-filtered on paper to obtain the liquid extract. Ultrasound energy was also recorded from the instrument.

A D-optimal design was generated and analyzed using MODDE version 13.0.2 (Sartorius Stedim Data Analytics AB, Umeå, Sweden) [26]. Three quantitative factors were studied at three levels each: extraction time (5, 12.5, and 20 min), ultrasound amplitude (20, 30, and 40%), and ethanol concentration (30, 50, and 70%). The design comprised 16 runs, including three center points performed as three independent extractions, and the run order was randomized. The TPC of each extraction run was measured in three analytical replicates, and the mean value of each run was used for model fitting. The response was the TPC of the liquid extracts (see Section 2.5). The optimum was determined with the MODDE optimizer, and the predicted optimum was verified in a subsequent confirmation experiment.

In order to obtain a significant mass for biological analyses, the optimized UAE was repeated 25 times (25 × 0.5, using 12.5 g of plant material), and the bulk or pooled extract was frozen and lyophilized to yield the optimized *E. cannabinum* dry extract (OpEU), which was stored in a desiccator at room temperature until analysis. The extraction yield (η) was calculated using Equation (1) and expressed as a percentage (%, *m*/*m*):

(1) $$\eta \;\%=100\times \frac{\mathrm{total mass of dry extract}\;(g)}{\mathrm{total mass of dry plant material}\;(g)}$$

### 3.5. Total Phenolic Content (TPC)

TPC was determined using the Folin–Ciocalteu method adapted to a 96-well microplate [14]. It was measured at two stages of the workflow and expressed on two different bases, which are not interchangeable, as follows:

(a) Design of experiments (DoE): TPC was measured directly in the liquid extract from each run and used as the response variable for optimization; results were expressed as mg gallic acid equivalents per gram of dried plant material (mg GAE/g dried plant).
(b) Optimized extract (OpEU). TPC of the lyophilized extract was determined separately and expressed as mg GAE per gram of dry extract (mg GAE/g dried extract); the same expression was reported in the antioxidant and chemical characterization results.

For the assay, 10 µL of sample was mixed with 100 µL of Folin–Ciocalteu reagent diluted 1:10 (*v*/*v*) with distilled water. The plate was shaken, and 80 µL of 1% (*w*/*v*) aqueous Na_2_CO_3_ was added. After 30 min of incubation at room temperature, protected from light, absorbance was read at 760 nm. The liquid extracts were analyzed after 10-fold dilution, and OpEU was analyzed as a 1 mg/mL solution in 30% ethanol. Concentrations were calculated from a gallic acid calibration curve (12 concentrations, with a range between 0.011 and 0.154 mg/mL). Samples were analyzed in three technical replicates, with the solvent blank being subtracted.

### 3.6. In Vitro Antioxidant Activity

The radical-scavenging activity of OpEU was evaluated with the DPPH and ABTS assays, adapted to 96-well microplates. In both assays, OpEU was dissolved in 30% ethanol (*v*/*v*) and serially diluted two-fold to obtain ten concentrations (100–0.2 µg/mL of extract). Ascorbic acid (DPPH) and Trolox (ABTS) were used as positive controls over the same concentration range. Each measurement was performed in three technical replicates. The percentage of radical scavenging (% I) was calculated using Equation (2):

(2) $$\%\;I=\frac{A_{C}-A_{S}}{A_{C}}\times 100$$

where A_C_ is the absorbance of the control (solvent and radical solution), and A_S_ is the absorbance of the sample. IC_50_ values were obtained by nonlinear regression of % I against the logarithm of the concentration and are expressed in mg/mL.

#### 3.6.1. Determination of DPPH Radical Scavenging Activity

The DPPH assay was adapted from a published method with minor modifications [20]. In brief, 50 µL of each OpEU dilution was mixed with 200 µL of a freshly prepared 0.1 mM methanolic DPPH solution, stored in an amber flask protected from light. The mixture was incubated for 30 min at room temperature in the dark, and absorbance was measured at 517 nm.

#### 3.6.2. Determination of ABTS Radical Scavenging Activity

The ABTS assay was adapted from a published method with minor modifications [27]. The ABTS radical cation (ABTS•^+^) was generated by mixing 7 mM ABTS with 2.45 mM potassium persulfate and keeping the mixture in the dark at room temperature for 12 h. Before the assay, the ABTS•^+^ stock solution was diluted with methanol until its absorbance at 734 nm was between 0.70 and 0.90 (measured in the microplate); this diluted solution was used as the working ABTS•^+^ solution. Then, 50 µL of each OpEU dilution was mixed with 200 µL of the working ABTS•^+^ solution and incubated for 30 min at room temperature (20 ± 2 °C) in the dark. Absorbance was measured at 734 nm.

### 3.7. Enzyme Inhibitory Activity

The inhibitory effects of OpEU on α-glucosidase, α-amylase, and pancreatic lipase were assessed in 96-well microplates. For this, OpEU was dissolved in 5% DMSO and serially diluted two-fold with 5% DMSO to give 16 concentrations in the range 4000–0.122 µg/mL, so that the DMSO content was constant at all tested concentrations. The final DMSO concentration in the well was 1.67% (*v*/*v*) in the α-glucosidase assay, 1% (*v*/*v*) in the α-amylase assay (during the enzymatic reaction), and 2% (*v*/*v*) in the lipase assay. Acarbose was used as the positive control for α-glucosidase and α-amylase, and orlistat for lipase, all prepared in the range of 3.33–333.33 µg/mL. In each assay, a solvent control containing 5% DMSO in place of the sample (same volume, and therefore the same final DMSO concentration) was included on every plate and used as the 0% inhibition reference for all tested concentrations, together with the corresponding sample blanks without enzyme. The IC_50_ values were obtained by nonlinear regression of % inhibition against the logarithm of concentration and are reported in mg/mL.

#### 3.7.1. α-Glucosidase Inhibitory Assay

To assess the inhibition of α-glucosidase, a slightly adjusted protocol as described earlier was applied [28]. In brief, 50 μL of different concentrations of the extract (4000–0.122 μg/mL; 0.04 to 1333.33 µg/mL per well) were mixed with 50 μL of the enzyme (0.75 U/mL in potassium phosphate buffer, pH = 6.8) and 50 μL of 0.5 mM *p*-nitrophenyl-α-D-glucopyranoside (pNPG). After a 15 min incubation at 37 °C, the absorbance was measured at a wavelength of 405 nm. The percentage of inhibition was calculated using Equation (3):

(3) $$\%I=100\times \frac{{(A}_{\mathrm{CB}}-A_{C})-(A_{\mathrm{SB}}-A_{S})}{A_{\mathrm{CB}}-A_{C}}$$

where A_C_ is the absorbance of the control, A_CB_ is the absorbance of the control blank, A_S_ represents the absorbance of the sample, and A_SB_ denotes the absorbance of the sample blank.

#### 3.7.2. α-Amylase Inhibitory Assay

To assess the inhibition of α-amylase, the assay was based on a published method [14]. In brief, 25 μL of different concentrations of the extract (4000–0.122 μg/mL; 0.02 to 800 µg/mL per well) was mixed with 50 µL of α-amylase in phosphate buffer (pH 6.9) and incubated for 10 min at 37 °C. Then, 50 µL of 0.05% starch solution was added, and the mixture was incubated for another 10 min at 37 °C. The reaction was stopped with 25 µL of 1 M HCl, and iodine–potassium iodide reagent was added. Absorbance was read at 630 nm. A control without enzyme was included. The percentage of inhibition was calculated as in Equation (4):

(4) $$\%I=100\times \frac{A_{S}-A_{E}}{A_{0}-A_{E}}$$

where A_S_ is the absorbance of the sample with enzyme, A_E_ that of the enzyme control without inhibitor, and A_0_ that of the control without enzyme.

#### 3.7.3. Pancreatic Lipase Inhibitory Assay

For the lipase inhibition test, the assay followed a published protocol [29]. In each well, 40 µL of sample (4000–0.122 μg/mL; 0.04 to 1600 µg/mL per well) was mixed with 40 µL of porcine pancreatic lipase type II (2.5 mg/mL) in Tris buffer (100 mM Tris-HCl, 5 mM CaCl_2_, pH = 7.0) and pre-incubated for 15 min at 37 °C. Then, 20 µL of 10 mM *p*-nitrophenyl butyrate (pNPB) was added, and the mixture was incubated for 10 min at 37 °C. Absorbance was read at 405 nm. The percentage of inhibition was calculated using Equation (3).

### 3.8. Antibacterial Activity

The in vitro antibacterial activity of the OpEU was evaluated against three Gram-positive bacterial strains (*Staphylococcus aureus* ATCC 25923, methicillin-resistant *Staphylococcus aureus*, MRSA, ATCC 43300, and *Enterococcus faecalis* ATCC 29212) and three Gram-negative bacterial strains (*Escherichia coli* ATCC 25922, *Klebsiella pneumoniae* ATCC 13883, and *Pseudomonas aeruginosa* ATCC 27853) using a broth microdilution method adapted from Coman et al. [30].

Specifically, 150 mg of lyophilized OpEU was dissolved in 5% DMSO to obtain an initial extract concentration of 10.18 mg/mL. In sterile 96-well microplates, 200 µL of this solution was placed in the first well and 100 µL of sterile distilled water in each following well. Two-fold serial dilutions were made by transferring 100 µL between adjacent wells with mixing, and 100 µL was discarded from the last well, leaving 100 µL per well. Thus, the first well contained 100 µL of OpEU at 10.18 mg/mL (in 5% DMSO), which, after the addition of 100 µL of inoculum, resulted in a final test concentration of 5.09 mg/mL (10.18 mg/mL × 100 µL/200 µL) and a final DMSO concentration of 2.5%.

Fresh cultures grown on Mueller–Hinton agar for 24 h at 37 °C were suspended in sterile saline, and the turbidity was adjusted to 0.5 McFarland. This suspension (≈1.5 × 10^8^ CFU/mL; 10 µL) was added to 9990 µL of double-strength Mueller–Hinton broth (2× MHB), giving ≈1.5 × 10^5^ CFU/mL, and 100 µL of the resulting inoculum was added to each well, giving a final volume of 200 µL and a final inoculum of ≈7.5 × 10^4^ CFU/mL. The final OpEU concentrations ranged from 5.09 to 0.002485 mg/mL (twelve two-fold dilutions: 5.09, 2.545, 1.2725, 0.63625, 0.31813, 0.15906, 0.07953, 0.03977, 0.01988, 0.00994, 0.00497 and 0.002485 mg/mL). The highest concentration contained 2.5% DMSO.

Levofloxacin was used as the positive antibacterial control and was evaluated using the same twofold broth microdilution procedure, using concentrations in µg/mL. A bacterial growth control (inoculum and 2× MHB without OpEU) and a sterility control (sterile distilled water and 2× MHB without inoculum) were included. A separate DMSO-only control was not included; however, the highest final DMSO concentration (2.5%, in the 5.09 mg/mL well) is below the concentrations generally reported to affect the growth of the tested reference strains, and growth was observed at this concentration for *E. faecalis* and *P. aeruginosa*. Each determination was performed in three technical replicates. The microplates were incubated for 24 h at 37 °C.

The minimum inhibitory concentration (MIC) was initially assessed by visually examining the wells for the absence of bacterial growth. Subsequently, 3 μL of 0.015% resazurin solution was added to each well, and the microplates were incubated for an additional 2–4 h at 37 °C. The MIC was defined as the lowest tested OpEU concentration at which resazurin retained its blue color, indicating the absence of detectable bacterial growth. When an inhibitory endpoint was not reached within the investigated concentration range, the MIC was reported as greater than the highest tested concentration (>5.09 mg/mL).

For determination of the minimum bactericidal concentration (MBC), all wells showing no visible bacterial growth were identified before the addition of resazurin. A 3 μL aliquot from each of these wells was inoculated onto Sheep Blood Agar plates, which were subsequently incubated for 18 h at 35 °C. The MBC was defined as the lowest tested OpEU concentration at which no bacterial growth was observed on Sheep Blood Agar. When bacterial growth was observed after subculturing from the highest tested concentration, the MBC was reported as >5.09 mg/mL.

### 3.9. UHPLC-UV Characterization of the OpEU Extract

The phenolic profile of OpEU was determined based on a previously described method [31,32]. For this, the extract was dissolved in a water–methanol mixture (1:9, *v*/*v*) at 1 mg/mL and filtered through a 0.22 µm filter. Twelve reference standards (eleutheroside B, catechin, chlorogenic acid, epicatechin, caffeic acid, vanillic acid, luteolin-7-*O*-glucoside, rutin, ellagic acid, taxifolin, sinapic acid and quercetin) were prepared as a mixed standard solution containing 200 µg/mL of each compound.

The analysis was carried out on a Flexar FX-10 UHPLC system (binary pump, in-line degasser, autosampler, column thermostat) with a Flexar FX-PDA detector (PerkinElmer Inc., Waltham, MA, USA). Separation was achieved on a Luna C18(2) column (150 × 4.6 mm, 3 µm; Phenomenex, Torrance, CA, USA) maintained at 35 °C [33], at a flow rate of 1 mL/min. The mobile phase consisted of 0.1% formic acid in water (A) and acetonitrile (B). The gradient was: 90% A (0–0.1 min); 90→20% A (0.1–20.1 min); 20% A (20.1–25.1 min); 20→90% A (25.1–26.1 min); and 90% A (26.1–30.1 min). The injection volume was 20 µL for both samples and standards.

Chromatograms were recorded at five wavelengths. For each compound, the wavelength with the best selectivity and sensitivity was used: 220 nm (luteolin-7-*O*-glucoside); 270 nm (eleutheroside B, caffeic acid, vanillic acid, rutin, ellagic acid); 280 nm (catechin, epicatechin, taxifolin); 324 nm (chlorogenic acid, sinapic acid); and 370 nm (quercetin). Compounds were identified by comparing retention times and UV spectra (220–370 nm) with those of the standards. Peak purity was not assessed; therefore, co-elution with other compounds cannot be excluded, and the reported contents should be regarded as tentative.

Quantification was based on a single calibration level (200 µg/mL of each standard). The method had been validated over a range of 3.125–200 µg/mL (linearity, R^2^, LoD, LoQ, precision, and accuracy; see Data Availability Statement). The content of each compound was calculated as mg/g freeze-dried extract.

### 3.10. Statistical Analysis

The experimental design and the regression model were modeled and evaluated in MODDE 13.0.2 (Sartorius Stedim Data Analytics AB, Umeå, Sweden) [26]. The model was fitted by multiple linear regression, and its quality was assessed with R^2^, adjusted R^2^, Q^2^, model validity, and reproducibility. The coefficients were assessed by ANOVA and 95% confidence intervals, and the residuals were examined with the model diagnostic plots (normal probability plot, residuals versus run order). The confirmation experiment was performed as three independent extractions (biological replicates). The in vitro assays (TPC, antioxidant, enzyme inhibition, and antibacterial activity) were performed on one pooled extract batch (OpEU) in three technical replicates, and the results are expressed as mean ± standard deviation (*n* = 3). The IC_50_ values were obtained by nonlinear regression of % inhibition against the logarithm of concentration.

## 4. Conclusions

A D-optimal design was used to optimize the ultrasound-assisted extraction of phenolic compounds from *Eupatorium cannabinum* L. aerial parts, with total phenolic content (TPC) as the only response. Ethanol concentration was the dominant factor, with TPC being the highest at 30% ethanol and increasing with extraction time, while amplitude had a smaller effect. The predicted optimum (30% ethanol, 20 min, 40% amplitude) was confirmed experimentally (31.32 mg GAE/g dried plant), although it lies at the boundary of the design space. The lyophilized extract (OpEU) contained 64.64 ± 2.64 mg GAE/g dry extract, and UHPLC-PDA tentatively indicated luteolin-7-*O*-glucoside and ellagic acid as the main targeted phenolics. In vitro, OpEU scavenged DPPH and ABTS radicals (IC_50_ 5.59 and 0.83 mg/mL), weakly inhibited α-glucosidase, α-amylase, and lipase (IC_50_ 2.7–3.6 mg/mL), and weakly inhibited four of six bacterial strains, only at 5.09 mg/mL, with no bactericidal effect.

These assays were exploratory, and all bioassays were performed on a single pooled extract batch obtained from plant material collected at one site and in one harvesting season (July 2022). The findings should therefore not be generalized to *E. cannabinum* as a species, nor to plant material from other populations, locations or harvesting seasons. Pyrrolizidine alkaloids, cytotoxicity, genotoxicity and other safety endpoints were not evaluated, so the results do not establish the safety, efficacy or suitability of OpEU for pharmaceutical, nutritional or health-related use. Targeted alkaloid profiling and toxicological evaluation are required before any application can be considered.

## Figures and Tables

**Table 1:** Design of experiments (DoE) matrix, total phenolic content (TPC, mg GAE/g of dried plant, mean ± standard deviation of three technical replicates of each extraction run), and the energy input for each experimental run. Runs N9–N11 represent the center points, performed as three independent extractions.

Sample Code	Run Order	EtOH (%)	Extraction Time (min)	Amplitude (%)	TPC (mg GAE/g)	Energy (Ws)
N1	1	30	5	20	20.79 ± 0.47	43
N12	2	70	5	20	13.71 ± 0.5	40
N2	3	30	20	20	26.89 ± 1.47	185
N13	4	70	20	20	11.74 ± 0.48	216
N3	5	30	5	40	23.75 ± 0.89	683
N14	6	70	5	40	12.51 ± 0.39	649
N4	7	30	20	40	30.72 ± 1.38	2399
N15	8	70	20	40	16.12 ± 0.61	2493
N5	9	30	5	30	26.6 ± 0.59	403
N6	10	30	12.5	20	26.35 ± 0.94	94
N16	11	70	12.5	20	11.41 ± 0.26	1006
N7	12	50	5	20	26.01 ± 0.99	47
N8	13	50	20	20	20.25 ± 0.47	165
N9	14	50	12.5	30	22.32 ± 0.65	1034
N10	15	50	12.5	30	23.55 ± 0.38	1090
N11	16	50	12.5	30	20.25 ± 0.10	890

**Table 2:** TPC-based optimal UAE conditions and model-predicted and experimentally measured TPC values, expressed as mean ± SD of three independent extractions (biological replicates, *n* = 3).

Parameter	TPC (mg GAE/g Plant)
Optimization goal (minimum/target/maximum)	25/30/35
Model-predicted	30.27
Experimentally measured	31.32 ± 0.58
Prediction accuracy (%) *	103.47 ± 1.93

* Calculated for each of the three independent confirmation extractions as 100 × (measured TPC/predicted TPC) and expressed as mean ± SD (*n* = 3). The predicted TPC (30.27 mg GAE/g dried plant) is a single model-derived value; the SD reflects only the variability between the confirmation extractions, not uncertainty of the model prediction. TPC: total phenolic content; GAE: gallic acid equivalents.

**Table 3:** Total phenolic content and in vitro antioxidant activity of the TPC-optimized *E. cannabinum* extract (OpEU), measured on the lyophilized extract. Values are expressed as mean ± SD (*n* = 3 technical replicates from one pooled extract batch).

Assay	OpEU (Mean ± SD)	95% CI
TPC (mg GAE/g of dried extract)	64.64 ± 2.64	58.08–71.20
DPPH (IC_50_, mg/mL of resolubilized extract)	5.59 ± 0.68	3.90–7.28
ABTS (IC_50_, mg/mL of resolubilized extract)	0.83 ± 0.11	0.56–1.10

Abbreviations: TPC: total phenolic content; GAE: gallic acid equivalents; DPPH: 2,2-diphenyl-1-picrylhydrazyl; ABTS: 2,2′-azino-bis(3-ethylbenzothiazoline-6-sulfonic acid); IC_50_: concentration of extract required to scavenge 50% of the radicals; CI: confidence interval. The 95% CIs were calculated from the three technical replicates (Student’s *t* distribution, df = 2).

**Table 4:** IC_50_ values for the inhibition of α-glucosidase, α-amylase, and pancreatic lipase by the TPC-optimized *E. cannabinum* extract (OpEU) and the corresponding positive controls. Values are expressed as mean ± SD (*n* = 3 technical replicates from one pooled extract batch).

Enzymatic Assay	Sample and Standard Compound	IC_50_ (mg/mL)
α-Glucosidase	OpEU	3.031 ± 0.095
	Acarbose	0.155 ± 0.021
α-Amylase	OpEU	3.553 ± 0.334
	Acarbose	0.021 ± 0.001
Lipase	OpEU	2.665 ± 0.356
	Orlistat	0.068 ± 0.002

Note: IC_50_ is the concentration required to inhibit 50% of the measured enzyme activity under the experimental assay conditions. Acarbose was used as the positive control for the α-glucosidase and α-amylase assays, whereas orlistat was used as the positive control for the pancreatic lipase assay.

**Table 5:** Minimum inhibitory (MIC) and minimum bactericidal (MBC) concentrations of OpEU and the MIC of levofloxacin.

Bacterial Strain	OpEU		Levofloxacin
	MIC (mg/mL)	MBC (mg/mL)	MIC (μg/mL)
*S. aureus* ATCC 25923	5.09	>5.09	0.25
MRSA ATCC 43300	5.09	>5.09	0.125
*E. faecalis* ATCC 29212	>5.09	>5.09	0.50
*E. coli* ATCC 25922	5.09	>5.09	0.03
*K. pneumoniae* ATCC 13883	5.09	>5.09	0.03
*P. aeruginosa* ATCC 27853	>5.09	>5.09	0.50

Note: >5.09 mg/mL: no inhibitory or bactericidal endpoint within the tested range (0.0025–5.09 mg/mL). *S. aureus*: *Staphylococcus aureus*; MRSA: Methicillin-Resistant *Staphylococcus aureus*; *E. faecalis*: *Enterococcus faecalis*; *E. coli*: *Escherichia coli*; *K. pneumoniae*: *Klebsiella pneumoniae*; *P. aeruginosa*: *Pseudomonas aeruginosa*. Final results are presented as the mean of three technical replicates from one pooled extract batch.

**Table 6:** Phenolic compounds identified in OpEU by UHPLC-PDA, expressed as mg/g dry extract.

Peak No.	Retention Time (min)	Compound Name	Amount (mg/g Dried Extract)	Wavelength (nm)
1	4.84	Eleutheroside B	<LoQ	270
2	4.96	Chlorogenic acid	2.253	324
3	5.03	(+)-Catechin	2.023	280
4	5.78	(−)-Epicatechin	<LoQ	280
5	6.00	Caffeic acid	<LoQ	270
6	6.12	Vanillic acid	<LoQ	270
7	6.78	Rutin	8.326	270
8	6.90	Luteolin-7-*O*-glucoside	67.389	220
9	7.24	Ellagic acid	28.612	270
10	7.98	Sinapic acid	<LoQ	324
11	8.05	Taxifolin	<LoQ	280
12	10.59	Quercetin	8.726	370

Note: LoQ: limit of quantification.

## Data Availability

The data supporting the findings of this study are included in the article and in the Supplementary Materials (Tables S1–S3, Figure S1). The raw data, including the DoE matrix, raw absorbance readings, model output, chromatographic peak table, dose–response data, and antibacterial results, are openly available at: https://bit.ly/RawDataPlants4515039 (accessed on 19 October 2026).

## Supplementary Materials

The following supporting information can be downloaded at: https://www.mdpi.com/article/10.3390/plants15213277/s1. Table S1: Raw experimental design dataset, used for DoE-based modeling and RSM. Response (TPC) was reported as mean value ± standard deviation of three technical replicates (*n* = 3); Table S2: Coefficient list (Scaled and centered—SC) with the corresponding statistical analysis; Table S3: ANOVA table for TPC as response; Figure S1: UPLC-PDA chromatograms of the optimized *Eupatorium cannabinum* extract (OpEU) recorded at the five preferential wavelengths used for compound identification and quantification: (A) 220 nm, (B) 270 nm, (C) 280 nm, (D) 324 nm, and (E) 370 nm.
