# Supplementary material for: Total Phenolic Content-Based Optimization of Ultrasound-Assisted Extraction from Eupatorium cannabinum L. Aerial Parts and In Vitro Evaluation of the Resulting Extract
Source: Plants (Basel). 2026 Oct 23;15(21):3277. doi: 10.3390/plants15213277 (PMC13765911; doi:10.3390/plants15213277)
Supplement: Supplementary file 1 [file plants-15-03277-s001.zip › plants-4515039-supplementary.pdf]

Supplementary material

Total Phenolic Content-Based Optimization of Ultrasound-Assisted Extraction from *Eupatorium cannabinum* L. Aerial Parts and *In Vitro* Evaluation of the Resulting Extract

Table S1. Raw experimental design dataset, used for DoE-based modelling and RSM. Response (TPC) was reported as mean value ± standard deviation of three technical replicates (n = 3).

Sample Code	Run Order	EtOH (%)	Extraction Time (min)	Amplitude (%)	TPC (mg GAE/mL extract)	TPC (mg GAE/g plant)	Energy (Ws)
N1*	1	30	5	20	1.05 ± 0.02	20.79 ± 0.47	43
N12	2	70	5	20	0.68 ± 0.02	13.71 ± 0.50	40
N2	3	30	20	20	1.34 ± 0.07	26.89 ± 1.47	185
N13	4	70	20	20	0.59 ± 0.02	11.74 ± 0.48	216
N3	5	30	5	40	1.19 ± 0.04	23.75 ± 0.89	683
N14	6	70	5	40	0.62 ± 0.02	12.51 ± 0.39	649
N4	7	30	20	40	1.55 ± 0.07	30.72 ± 1.38	2399
N15	8	70	20	40	0.8 ± 0.03	16.12 ± 0.61	2493
N5	9	30	5	30	1.34 ± 0.03	26.6 ± 0.59	403
N6	10	30	12.5	20	1.32 ± 0.05	26.35 ± 0.94	94
N16	11	70	12.5	20	0.57 ± 0.01	11.41 ± 0.26	1006
N7	12	50	5	20	1.3 ± 0.05	26.01 ± 0.99	47
N8	13	50	20	20	1.01 ± 0.02	20.25 ± 0.47	165
N9	14	50	12.5	30	1.13 ± 0.03	22.32 ± 0.65	1034
N10	15	50	12.5	30	1.18 ± 0.02	23.55 ± 0.38	1090
N11	16	50	12.5	30	1.00 ± 0.00	20.25 ± 0.10	890

* Experimental run excluded from modelling (identified as an analytical outlier).

Table S2. Coefficient list (Scaled and centered – SC) with the corresponding statistical analysis.

TPC	Coefficient	Coeff. SC	Std. Err.	P	Conf. int(±)
**Constant**		22.0055	0.744413	9.93561e-08	1.82153
**EtOH**	X_1	-7.01995	0.449066	4.34008e-06	1.09883
**Time**	X_2	0.497283	0.449066	0.310541	1.09883
**Amplitude**	X_3	0.221661	0.457425	0.645152	1.11929
**EtOH*EtOH**	X_1^2	-2.84974	0.844015	0.0149237	2.06524
**Time*Time**	X_2^2	1.40025	0.844015	0.14818	2.06524
**EtOH*Time**	X_1X_2	-0.388248	0.50782	0.473532	1.2426
**EtOH*Amp**	X_1X_3	0.569235	0.485795	0.285712	1.18871
**Time*Amp**	X_2X_3	2.1608	0.485795	0.00433861	1.18871

Note: The model equation based on the coefficient estimates (scaled and centered) can be expressed as:

$$Y = 22 - 7.02X_1 + 0.50X_2 + 0.22X_3 - 2.85X_1^2 + 1.4X_2^2 - 0.39X_1X_2 + 0.60X_1X_3 + 2.16X_2X_3$$

Table S3. ANOVA table for TPC as response.

TPC	DF	SS	MS (variance)	F	p	SD
Total	15	7055.17	470.345			
Constant	1	6497.63	6497.63			
Total corrected	14	557.537	39.8241			6.31063
Regression	8	545.808	68.226	34.8991	**0.000**	8.2599
Residual	6	11.7297	1.95495			1.3982
Lack of Fit (Model error)	4	6.16196	1.54049	0.553362	**0.724**	1.24116
Pure error (Replicate error)	2	5.56774	2.78387			1.66849

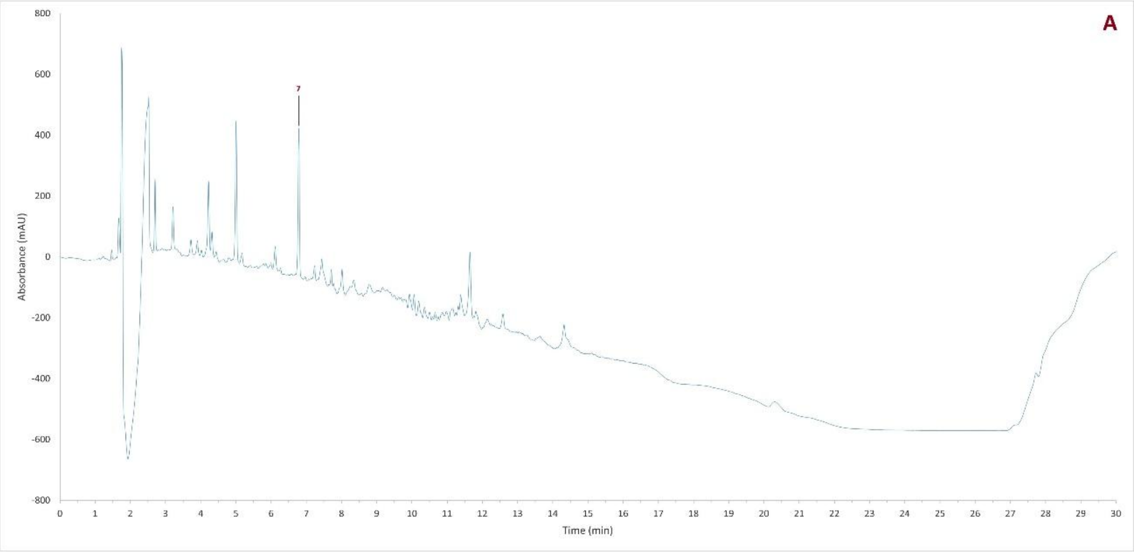
A
Absorbance (mAU)
Time (min)
7

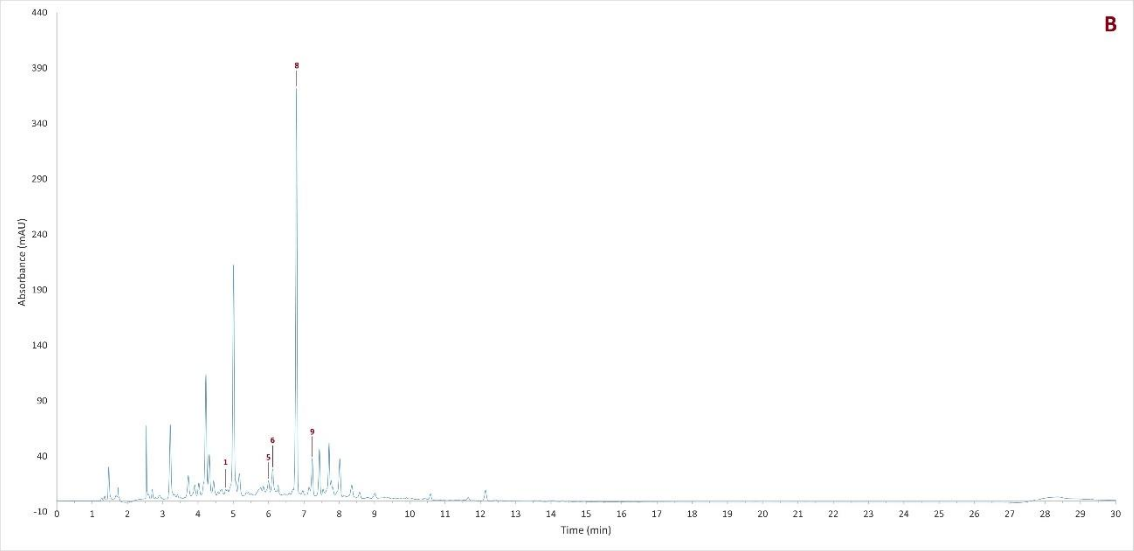
B
Absorbance (mAU)
Time (min)
1
5
6
8
9

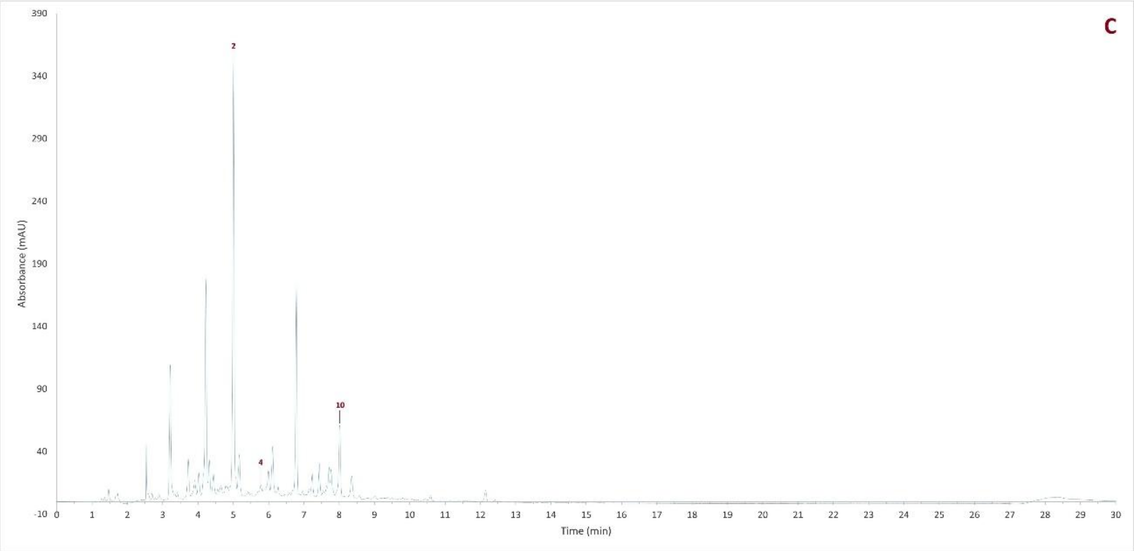
C
Absorbance (mAU)
Time (min)
2
4
10

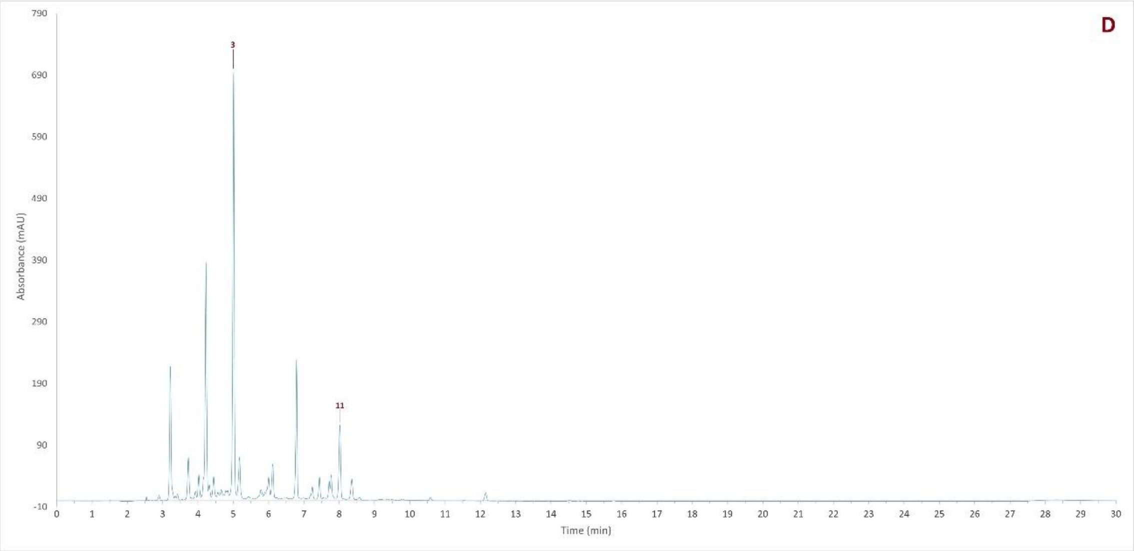

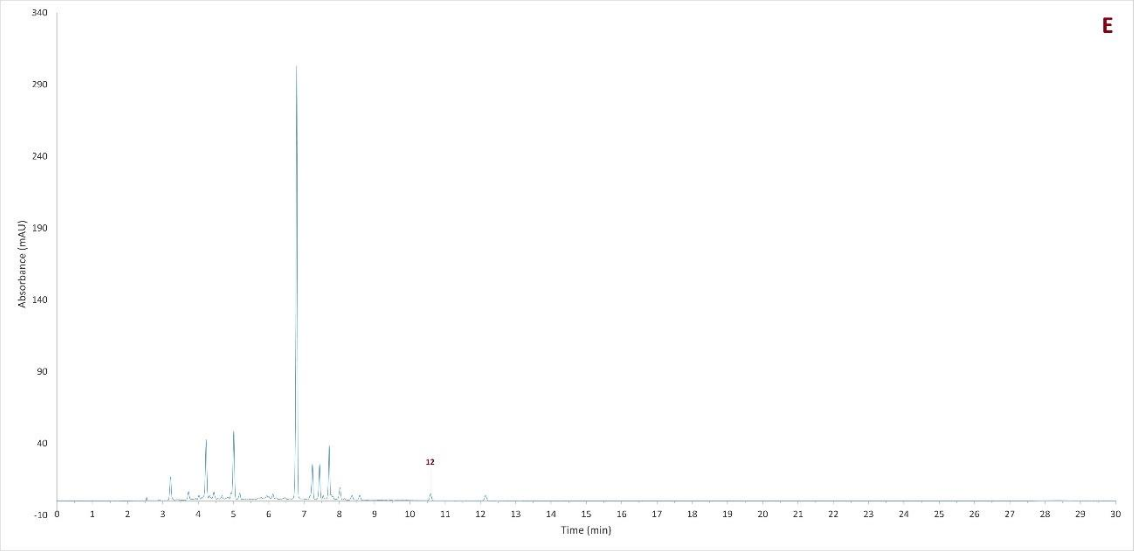

Figure S1. UPLC-PDA chromatograms of the optimized *Eupatorium cannabinum* extract (OpEU) recorded at the five preferential wavelengths used for compound identification and quantification: (A) 220 nm, (B) 270 nm, (C) 280 nm, (D) 324 nm, and (E) 370 nm. These chromatograms illustrate the detection profile of the identified phenolic constituents presented in Table 6 of the main manuscript.
